# Case report: A germline *CHEK1 c.613 + 2T>C* leads to a splicing error in a family with multiple cancer patients

**DOI:** 10.3389/fonc.2024.1380093

**Published:** 2024-04-15

**Authors:** Jun Qian, Min Peng, Yanan Li, Wei Liu, Xinwei Zou, Huafei Chen, Sujuan Zhou, Sheng Xiao, Jinhua Zhou

**Affiliations:** ^1^ Department of Gynecology and Obstetrics, The First Affiliated Hospital of Soochow University, Suzhou, China; ^2^ Molecular Genetics Laboratory, Suzhou Sano Precision Medicine Ltd., Suzhou, China; ^3^ Department of Pathology, Brigham and Women’s Hospital, Harvard Medical School, Boston, MA, United States

**Keywords:** *CHEK1*, germline mutation, splicing error, ovarian cancer, inherited cancer

## Abstract

**Background:**

Genome instability plays a crucial role in promoting tumor development. Germline mutations in genes responsible for DNA repair are often associated with familial cancer syndromes. A noticeable exception is the *CHEK1* gene. Despite its well-established role in homologous recombination, germline mutations in *CHEK1* are rarely reported.

**Case presentation:**

In this report, we present a patient diagnosed with ovarian clear cell carcinoma who has a family history of cancer. Her relatives include a grandfather with esophageal cancer, a father with gastric cancer, and an uncle with a brain tumor. The patient carried a typical genomic profile of clear cell carcinoma including mutations in *KRAS*, *PPP2R1A*, and *PIK3R1*. Importantly, her paired peripheral blood cells harbored a germline *CHEK1* mutation, *CHEK1 exon 6 c.613 + 2T>C*, which was also found in her father. Unfortunately, the *CHEK1* status of her grandfather and uncle remains unknown due to the unavailability of their specimens. Further evaluation via RT-PCR confirmed a splicing error in the *CHEK1* gene, resulting in truncation at the kinase domain region, indicative of a loss-of-function mutation.

**Conclusion:**

This case highlights a rare germline *CHEK1* mutation within a family with a history of cancer. The confirmed splicing error at the mRNA level underscores the functional consequences of this mutation. Documenting such cases is vital for future evaluation of inheritance patterns, clinical penetrance of the mutation, and its association with specific cancer types.

## 1 Introduction

DNA repair involves complex mechanisms and intricate pathways. Both checkpoint kinase 1 and 2 (CHEK1, CHEK2) play crucial roles in DNA repair and safeguarding genomic stability during the cell cycle (1). They serve as vital checkpoints that, when activated, temporarily halt the cell cycle, allowing time for DNA repair or triggering apoptosis when DNA damage becomes irreparable. Specifically, CHEK1 is activated by the DNA damage sensor known as the ataxia telangiectasia and Rad3-related protein (ATR) in response to single-strand DNA breaks during the S phase of the cell cycle. Subsequently, CHEK1 phosphorylates CDC25A and CDC25C, leading to the inhibition of cyclin-dependent kinases (CDKs) and cell cycle arrest (2, 3). CHEK2 is activated when double-strand DNA damage is detected by Ataxia-telangiectasia mutated (ATM) during the G1 phase of the cell cycle (4). The activated CHEK2 kinase phosphorylates and stabilizes p53 (ATM-CHEK2-P53 axis), leading to p21 expression (5–7). p21 is a potent CDK inhibitor that blocks the cell cycle progress (8).

Germline mutations in genes involved in DNA repair pathways are associated with genome instability and an increased risk of cancer development. For example, individuals with Li-Fraumeni syndrome are predisposed to various cancers, including leukemia, sarcomas, brain tumors, adrenocortical carcinoma, and other solid tumors, often manifesting at a young age (9). While *TP53* germline mutations are frequently associated with Li-Fraumeni syndrome, some individuals with TP53-negative Li-Fraumeni syndrome harbor germline mutations in *CHEK2* (10, 11). Germline mutation of *CHEK1*, however, is rarely documented in inherited cancers. This report presents the discovery of a novel *CHEK1* splicing mutation within a family with multiple cancer patients. This mutation results in a splicing error and a frameshift coding sequence alteration, confirming its inactivation nature.

## 2 Case presentation

**Figure 1:**
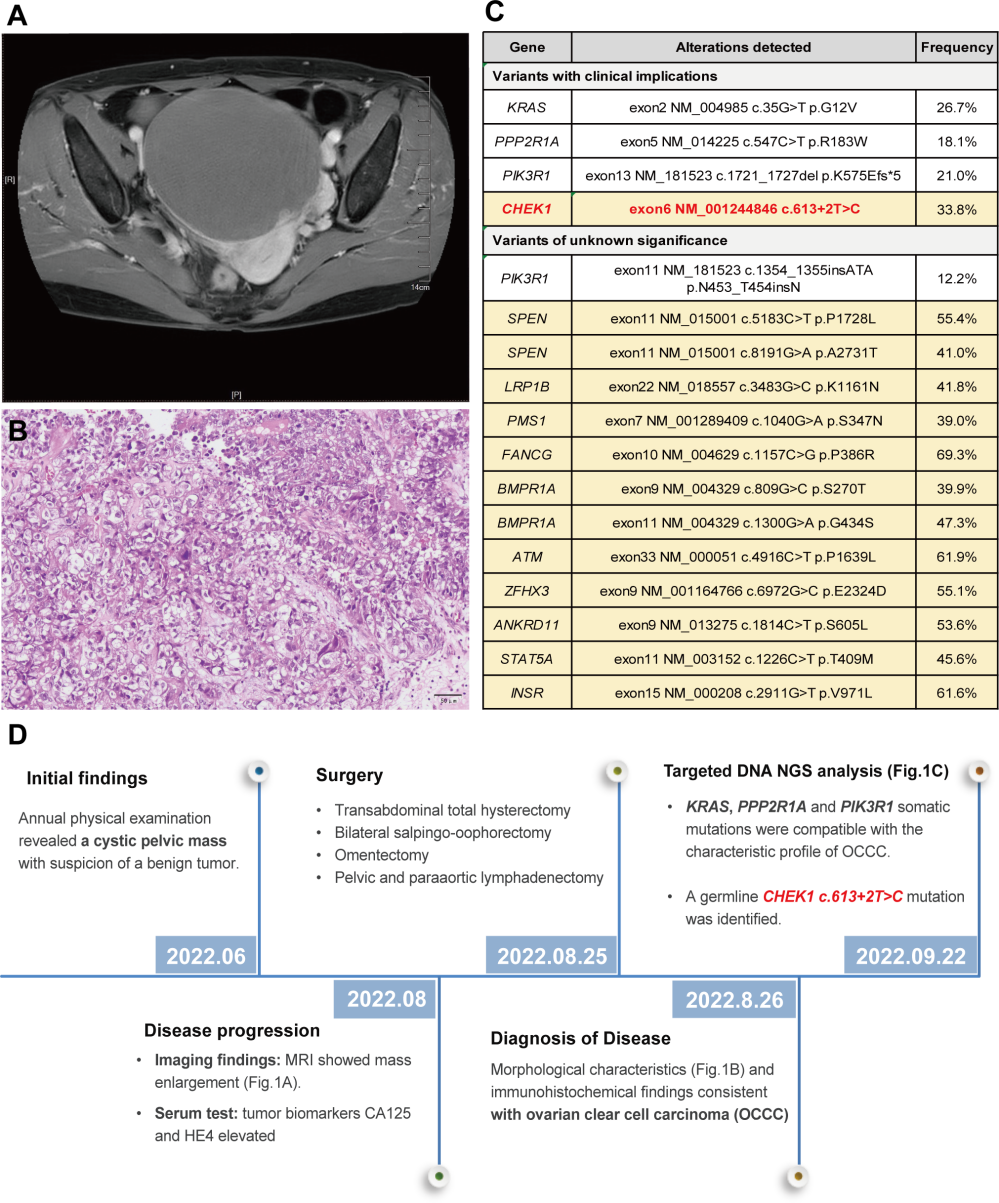
MRI revealed a pelvic cystic mass **(A)**; histological examination of the tumor demonstrated a clear cell morphology with papillary and focal solid growth patterns, consistent with the diagnosis of ovarian clear cell carcinoma (OCCC) **(B)**; Genomic profiling of the tumor exhibited a somatic mutation pattern consistent with the OCCC. Germline mutations, including *CHEK1*, were detected in the peripheral blood specimen (highlighted in yellow) **(C)**; and a timeline summarizing the key clinical events of the proband **(D)**.

During her annual physical examination, a 57-year-old female presented with pelvic effusion and a cystic mass measuring 119 × 126 × 100 mm, which exhibited nodular protrusions into the cavity as revealed by gynecological B-ultrasound. A benign ovarian tumor was suspected at the time. However, two months later, an MRI evaluation indicated a progressive enlargement of the mass (**Figure 1A**). Further serum examination showed elevated levels of tumor biomarkers: CA125 at 49.80 U/mL (normal range 0.1 ~35 U/ml) and HE4 at 3250 pmol/L (normal range <70 pmol/L for premenopause women and <140 pmol/L for post-menopause women). The patient underwent a transabdominal total hysterectomy, bilateral salpingo-oophorectomy, omentectomy, and pelvic and paraaortic lymphadenectomy. Hematoxylin and eosin (H&E) staining of formalin-fixed paraffin-embedded (FFPE) sections from the right ovarian appendage revealed a clear cell carcinoma with papillary and focal solid growth patterns (**Figure 1B**). Tumor cells contained clear cytoplasm, uniform nuclear atypia, prominent nucleoli, and occasional mitosis. Immunohistochemistry of the tumor cells was positive for ER (90% +), HNF1-β, PAX-8, p53(diffuse +), p16 (partial +), Ki-67 (30% +), and negative for PR, Vimentin, WT1, and Napsin A. Based on these findings, a diagnosis of ovarian clear cell carcinoma (OCCC) was established.

Targeted DNA NGS analysis of 638 cancer-related genes, combined with whole-genome single-nucleotide polymorphism (SNP) examination, was performed on both tumor and paired blood specimens. This analysis revealed somatic mutations, including *KRAS exon 2 c.35G>T* p.G12V (26.7%), *PPP2R1A exon 5 c.547C>T* p.R183W (18.1%), and *PIK3R1 exon 13 c.1721_1727del* p.K575Efs*5 (21%) (**Figure 1C**). These somatic mutations align with the characteristic profile of ovarian clear cell carcinoma. Additionally, a *CHEK1 exon 6 c.613 + 2T>C* mutation was identified in both the tumor and normal blood cells, confirming its germline origin. The timeline summarized the major clinical events of the patient (**Figure 1D**).

**Figure 2:**
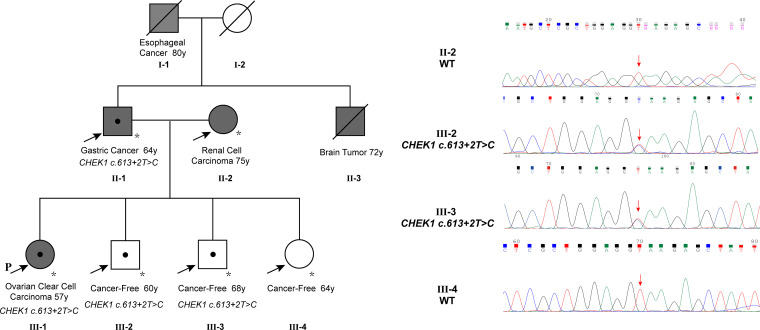
The family pedigree showed multiple members with cancer, including the proband with ovarian cancer at 57, the father with gastric cancer at 64, an uncle with a brain tumor at 72, the grandfather with esophageal cancer at 80, and the mother with renal cell carcinoma at 75. The *CHEK1* c.613 + 2T>C mutation was confirmed in the proband, the father, and two siblings. Note that the mutation in the father was validated using NGS instead of Sanger sequencing due to poor sequencing results from the PCR product, likely attributed to low DNA quality from the very old tumor block.

The patient reported a family history of cancer, spanning esophageal cancer in her grandfather, gastric cancer in her father, a brain tumor in her uncle, and renal cell carcinoma in her mother. Her grandfather (I-1) and uncle (II-3) were diagnosed with esophageal cancer and a brain tumor, respectively, based on imaging without biopsy, and both succumbed to the disease without undergoing surgery. Her father (II-1) had stage 2A gastric adenocarcinoma in 1995, which was successfully treated with surgery. The patient’s mother (II-2) underwent left-sided renal cancer surgery in 2007, with a postoperative histopathologic diagnosis of clear cell carcinoma. As of genetic counseling in February 2023, her other siblings had no reported history of cancer. To assess the presence of the *CHEK1* mutation in family members, a PCR reaction was performed to amplify the DNA fragment containing the mutation site using gene-specific primers (CHEK1_DNA_F: GCTCCAGAACTTCTGAAGAGAAG; CHEK1_DNA_R: GCTGCAGTGAGCTATAACAGC). The PCR product was subjected to direct Sanger sequencing. These studies detected the same *CHEK1* mutation in her father, who was diagnosed with gastric cancer at the age of 64, and two siblings, both of whom remained tumor-free at the ages of 60 and 68, respectively. Unfortunately, no specimens were available to evaluate the mutation status of her deceased grandfather, who was diagnosed with esophageal cancer at the age of 80, and her uncle, who was diagnosed with a brain tumor at the age of 72 (**Figure 2**).

**Figure 3:**
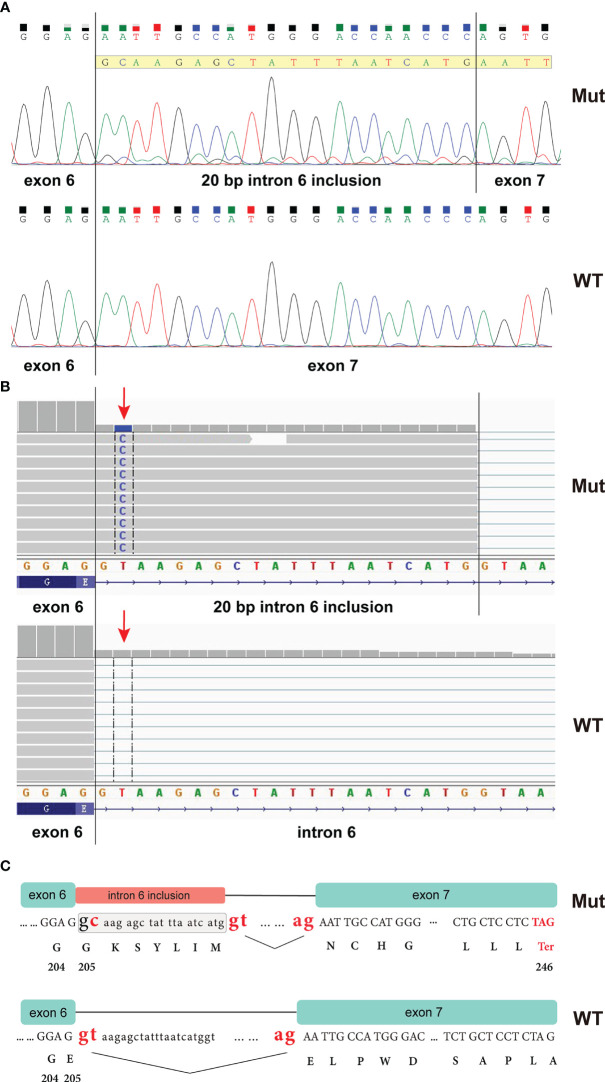
RT-PCR analysis of peripheral blood from the proband revealed that the *CHEK1 c.613 + 2T>C* mutation caused a splicing error, resulting in the inclusion of a 20 bp intronic sequence in the *CHEK1* transcript **(A)**; an RNA NGS assay demonstrated a similar aberrant *CHEK1* transcript due to the mutation at the splicing site (arrow) **(B)**; and an ideogram illustrated the wild-type splicing donor and acceptor sites of *CHEK1* intron 6, along with the alternative splicing donor and acceptor sites from the mutated allele. This alternative splicing event leads to the formation of a premature stop codon **(C)**.

The *CHEK1* exon 6 *c.613 + 2T>C* mutation was not documented in any cancer databases or in gnomAD. To determine whether this mutation impacts RNA splicing, an RT-PCR analysis was performed using peripheral blood from the proband. This assay amplified a fragment encompassing the splicing sites of exons 6 and 7 (CHEK1_RNA_F: 5’-GCTCCAGAACTTCTGAAGAGAAG; CHEK1_RNA_R: 5’-CTTGCTGATGGATTCTCAACT). Sanger sequencing of this PCR product confirmed that the *CHEK1 c.613 + 2T>G* mutation disrupted the donor splice site of exon 6, leading to the utilization of an alternative splice site located 20 bp downstream of exon 6 (**Figure 3A**). A similar aberrant *CHEK1* transcript was confirmed by a RNAseq analysis (**Figure 3B**). This resulted in a reading frame shift after amino acid residue 204 of CHEK1 and the presence of a premature translational stop codon at 41 amino acid residues downstream of the mutated nucleotide (truncated CHEK1 p. E205Gfs*41) (**Figure 3C**).

## 3 Discussion

CHEK1 is a serine/threonine kinase composed of an N-terminal kinase domain (amino acid residues 8-265) and a C-terminal regulatory domain (residues 317-476). The C-terminal regulatory domain of CHEK1 is self-inhibitory, interacting with the N-terminal to block the kinase activity of CHEK1 (12, 13). Upon encountering DNA damage or replication stress signals, the ATR kinase becomes activated, phosphorylating multiple serine/glutamine residues within the C-terminal of CHEK1, especially Ser317 and Ser345 residues. This phosphorylation event leads to the dissociation of the N- and C-terminal structural domains, activating CHEK1 (13–16). It has been demonstrated that the catalytic activity of the N-terminal kinase domain alone is significantly higher than that of the full-length CHEK1 protein *in vitro* (17). However, the integrity of the C-terminal regulatory domain is crucial for the proper *in vivo* functioning of the CHEK1 protein. In a study conducted by Ning et al. (12), the effects of various CHEK1 truncating variants on cell cycle regulation were investigated. The CΔ368 variant (residues 368-476 truncation) exhibited higher catalytic activity compared to full-length CHEK1, while CΔ288 (residues 288-476 truncation) had nearly lost its entire catalytic activity. Therefore, the C-terminal regulatory domain of CHEK1 contains not only inhibitory elements but also essential positive regulatory elements for catalytic activity (13–16). The *CHEK1 c.613 + 2T>G* splice variant reported in this study was predicted to generate a truncating protein p.E205Gfs*41, resulting in a partial loss of the N-terminal kinase domain (residues 205-265) and the complete deletion of the C-terminal, thus predicting a loss-of-function mutation.

The role of CHEK1 in DNA repair through homologous recombination is well-established. For instance, knocking down CHEK1 has been shown to result in deficient homologous recombination repair, confirming its function as a BRCA-like tumor suppressor (18). In addition, CHEK1 heterozygous deletion has been observed to accelerate tumorigenesis in WNT1 transgenic oncogenic mice compared to wild-type mice (19), as well as in chemically induced skin papilloma formation (20). During the early stages of tumorigenesis, partial deletion of CHEK1 has been linked to genomic instability, which in turn accelerates tumor development (19, 21, 22). However, the status of CHEK1 as a bona fide tumor suppressor remains controversial. CHEK1 is generally overexpressed in various cancers, including ovarian, breast (23), cervical cancer, and brain cancers (24), where tumor cells gain a survival advantage by enhancing checkpoints to facilitate DNA damage repair (25, 26). Elevated CHEK1 levels (both protein and mRNA) have been associated with chemoresistance (27–29), and many clinical trials are evaluating CHEK1 inhibitors in combination with chemotherapy (3, 26). On the other hand, loss-of-function variants have only been identified in a few cancers, such as gastric (30), colorectal (31, 32), and endometrial cancers (31, 33) with MSI-H features. Therefore, while germline CHEK1 mutations with loss-of-function potential may contribute to genome instability, cell cycle deregulation, and tumor development, somatic tumor cells with functional CHEK1 or gain-of-function CHEK1 alterations may assist in tumor cell survival by promoting lethal DNA damage repair.

Epithelial ovarian cancer is categorized into “Type I” and “Type II” subtypes (34). Our proband was diagnosed with OCCC, which is a Type I ovarian cancer and typically follows a relatively indolent course, often progressing through multiple steps from atypical hyperplasia, precancerous lesions, and borderline tumors (34, 35). Hereditary ovarian cancer associated with *BRCA1/2* mutations usually manifests as Type II tumors, characterized by rapid disease progression and high aggressiveness (35–38). However, OCCC is rarely linked to germline *BRCA1/2* mutations (39, 40). OCCC is hypothesized to arise from benign ectopic endometrial tissue on the ovary (41, 42), with approximately 50% to 74% of OCCC cases associated with endometriosis. It is suggested that the inflammatory and oxidative stress responses induced by endometriosis contribute to DNA damage and the development of malignancy (42). The most frequent genomic alterations in OCCC involve somatic mutations in *ARID1A* and *PIK3CA*, with loss of *ARID1A* function considered one of the earliest events, occurring in atypical endometriosis (43–45), and contributing to genomic instability. However, *ARID1A* loss alone is insufficient for tumor formation, typically occurring concomitantly with activation of the PI3K/AKT/mTOR pathway. *ARID1A* and *PIK3CA* alterations coexist in 20% to 56% of OCCC cases (46). Mouse models harboring both *ARID1A* and *PIK3CA* mutations develop tumors that phenotypically and molecularly resemble human OCCC (47), confirming the pivotal role of these pathways in OCCC pathogenesis. Our proband had no history of endometriosis or *ARID1A* mutation. We hypothesize that germline haploinsufficiency of CHEK1, as an alternative early genomic instability event, contributed to OCCC oncogenesis by cooperating with PI3K pathway activation. Notably, in the DNA damage response signaling, ARID1A interacts with ATR and is recruited to double-strand breaks to sustain DNA damage signaling (48). Defective ARID1A may impair DNA damage-induced ATR activation and its downstream signaling, particularly involving CHEK1 (48). Thus, functional defects of the CHEK1 may partially overlap with ARID1A in initiating events within this pathway.

While the *CHEK1* gene is included in some commercial NGS panels designed for assessing hereditary cancer risk (49), the available data regarding germline *CHEK1* mutations and cancer risk are limited. In a study involving 48 women with inherited ovarian cancer lacking *BRCA1* or *BRCA2* mutations, a *CHEK1 exon 7 c.1564-1565insA* frameshift mutation was identified (50). Additionally, in a group of 246 prostate cancer patients with a cancer history recommended for germline mutation testing by NCCN guidelines, a germline *CHEK1* “stop-gain” mutation was detected (51). However, neither study provided comprehensive mutation tracking within family members. The rarity of germline *CHEK1* mutations may underscore its significance in embryonic and organizational development. In mouse models, homozygous deletion of *CHEK1* resulted in embryonic death (19, 52), whereas mice with heterozygous loss of *CHEK1* were viable but exhibited haploinsufficiency, showing increased accumulation of DNA damage, cell cycle dysregulation, increased spontaneous cell death, and defects in tissue development (19, 53, 54). Moreover, CHEK1 plays a crucial role in maintaining functional hematopoiesis, as CHEK1 haploinsufficiency leads to anemia and abnormal erythropoiesis in mice (55). Chemical inhibition of CHEK1 induced hematopoietic stem cell and progenitor cell death in both mice and humans (56). Recent studies have reported several germline heterozygous mutations occurring in the C-terminal regulatory domain of *CHEK1*, including three missense variants (R379Q, R420K, and R442Q) and a truncating variant (F441fs*). These mutations were demonstrated to be gain-of-function, with increased kinase activity of CHEK1 causing arrested fertilized ovum division and resulting in infertility in human females (57–59).

In summary, we report a rare germline inactivation mutation of *CHEK1* in a family with a history of cancer, and we confirmed that this mutation led to splicing errors at the mRNA level. Although data on the prevalence of *CHEK1* germline mutations in inherited cancer is limited, along with their clinical penetrance and association with specific cancer types, documenting these mutations holds significant value for future assessments and conclusions.

## Data availability statement

The raw data supporting the conclusions of this article will be made available by the authors, without undue reservation.

## Ethics statement

Ethical review and approval were not required for the study on human participants in accordance with the local legislation and institutional requirements. Written informed consent to participate in this study was provided by the individual(s) and minor(s)’ legal guardian/next of kin to participate in this study. Written informed consent was obtained from the individual(s), and minor(s)’ legal guardian/next of kin, for the publication of any potentially identifiable images or data included in this article.

## Author contributions

JQ: Data curation, Writing – original draft, Writing – review & editing, Investigation. MP: Writing – review & editing, Writing – original draft, Investigation, Validation. YL: Investigation, Writing – original draft. WL: Software, Validation, Writing – review & editing, Data curation. XZ: Writing – review & editing, Data curation, Validation. HC: Methodology, Resources, Writing – review & editing. SZ: Methodology, Resources, Writing – review & editing. SX: Conceptualization, Supervision, Writing – review & editing, Project administration. JZ: Conceptualization, Project administration, Supervision, Writing – review & editing.
